# Inherited common variants in mitochondrial DNA and invasive serous epithelial ovarian cancer risk

**DOI:** 10.1186/1756-0500-6-425

**Published:** 2013-10-22

**Authors:** Madalene A Earp, Angela Brooks-Wilson, Linda Cook, Nhu Le

**Affiliations:** 1 Canada’s Michael Smith Genome Sciences Center, BC Cancer Agency, Vancouver, BC, Canada; 2 Department of Biomedical Physiology and Kinesiology, Simon Fraser University, Burnaby, BC, Canada; 3 Epidemiology and Biostatistics, Department of Internal Medicine, NM Health Sciences Center, University of New Mexico, MSC 10 5550, 1 University of New Mexico, Albuquerque, NM 87131-0001, USA; 4 Department of Population Health Research, Division of Cancer Care, Alberta Health Services, 2210-2nd St SW, Calgary, AB T2C 3S3, Canada; 5 Department of Community Health Sciences, Faculty of Medicine, University of Calgary, 3330 Hospital Drive N.W, Calgary, AB T2N 4N2, Canada; 6 Cancer Control Research, BC Cancer Agency, Vancouver, BC G12 0YN, Canada; 7 Genome Sciences Centre, BC Cancer Research Centre, 675 West 10th Avenue, Vancouver BC V5Z 1L3, Canada

**Keywords:** Mitochondrial DNA, Single nucleotide polymorphism, Epithelial ovarian cancer, Serous ovarian cancer, Haplogroup, Heritable risk, European

## Abstract

**Background:**

Mitochondria are the site of oxidative phosphorylation, a process which generates reactive oxygen species (ROS). Elevated ROS levels can lead to oxidative stress, a cellular state implicated in carcinogenesis. It is hypothesized that alternations in mitochondrial (MT) DNA, including heritable MT single nucleotide polymorphisms (MT-SNPs), have the potential to change the capacity of MT function, leading to increased oxidative stress and cancer risk. We investigated if common MT-SNPs and/or haplogroups and are associated with invasive serous ovarian cancer (OvCa) risk.

**Methods:**

A panel of 64 MT-SNPs designed to tag all common variation in the European MT genome (minor allele frequency (MAF) >1%, r^2 >0.8) was genotyped in study participants of European descent using the Sequenom MassARRAY iPlex Gold® system (Sequenom Inc, CA, USA). Invasive serous OvCa cases (n = 405) and frequency age-matched controls (n = 445) were drawn from a population-based case-control study of OvCa in western Canada. Binary logistic regression was used to estimate the odds ratio (OR) and 95% confidence intervals (C.I.) for carriage of the minor versus major allele by case-control status. MitoTool was used to test the relationship between European haplogroup status and case-control status using Fisher’s exact test.

**Results:**

The most significant disease-SNP association was for rs2857285, a synonymous MT-SNP in *ND4* (OR = 4.84, 95% CI: 1.03–22.68, *P* = 0.045). After adjustment for multiple testing using a Bonferroni correction of the Type 1 error this MT-SNP was not significant. No other MT-SNP had a *P-value* < 0.05. European haplogroup status was not associated with case status. Most MT-SNPs (73%) genotyped had a MAF <5%.

**Conclusion:**

Common European MT-SNPs (MAF > 5%) and haplogroups were not associated with invasive serous OvCa risk in this study; however, most European MT-SNPs have a low MAF (<5%), which we were underpowered to adequately assess. Larger studies are needed to clarify the role of low MAF MT-SNPs (MAF < 5%) in invasive serous OvCa risk.

## Background

Recent genome-wide association studies (GWAS) have revealed several loci associated with epithelial ovarian cancer (OvCa) risk [1-3]; however, much of the heritable risk for this disease remains unexplained. The mitochondrial (MT) genome is an underexplored potential source of OvCa risk. This small (16.6 kb), circular, maternally-inherited genome encodes 37 genes, all of which are involved in oxidative phosphorylation (OXPHOS), the process by which humans produce most of their energy. A byproduct of OXPHOS is the production of reactive oxygen species (ROS), unstable compounds which can generate free radicals. Cells are equipped to manage a certain level of free radical production; however, if threshold levels are exceeded, a state of oxidative stress occurs. In this state, lipids, proteins and nucleic acids are subject to attack by ROS [4]. It is hypothesized that alternations in MT DNA, including heritable single nucleotide polymorphisms (SNPs), have the potential to change MT function and lead to increased oxidative stress and cancer risk [4,5]. We investigated this possibility.

The ovarian surface epithelium (OSE), the site of most OvCa, is a unique microenvironment in the body. With each ovulation the OSE experiences a trauma where the mature follicle ruptures from the ovary. At this site there are inflammatory mediators and sub-lethal concentrations of ROS ([6,7]). Heritable MT-SNP variation could act synergistically with the unique conditions of the OSE, for example, elevated levels of somatic MT-DNA mutation incurred due to ROS, and explain why OvCa risk, not all cancer risk, might be elevated by germ line MT-SNPs. In connection with this, recent work has suggested that certain MT-DNA haplogroups have tissue-specific and stage-specific roles in modulating cancer development [8]. Human MT-DNA haplogroups are defined by unique combinations of MT-DNA polymorphisms, along discrete maternal lineages due to genetic drift and/or selection for MT function (return to original). These mutations must have occurred in oocyte mitochondria as only these MT genomes are inherited.

Two studies have examined the potential association between heritable MT-SNPs and OvCa risk [9,10]. Histology-specific analyses have not yet been done and to our knowledge no studies have examined the association between MT-DNA haplogroup and OvCa risk. Therefore, we evaluated the contribution of heritable MT-SNPs and haplogroups to the risk of developing serous OvCa in European women. Somatic MT-DNA mutations, for example, those arising in the OSE, were not assessed by this study.

## Methods

### Subjects

Study participants were drawn from the Ovarian Cancer in Alberta and British Columbia (OVAL-BC) study. OVAL-BC is a population-based case control study started in 2002 and completed recruitment in 2011. OVAL-BC aims to investigate ovarian cancer risk associated with environmental and genetics factors, along with their interactions. Eligible OVAL-BC cases had incident, histologically confirmed OvCa identified from the population-based provincial cancer registries in British Columbia (BC) and Alberta (AB). Eligible control women were identified from provincial health care enrollment rosters (BC and AB) or from a province-wide mammography program (BC after 2005). Epidemiological data was collected through questionnaire/interview from participants who also provided blood or saliva samples for DNA. OVAL-BC has a total of 1578 cases and 2222 controls, with 64.9% and 55.6% response rates respectively. The present analysis is based on the study population from BC only. This study was approved by the joint Clinical Research Ethics Board of the BC Cancer Agency and the University of BC. All subjects gave written informed consent.

Cases selected for this study (n = 405) were European women diagnosed with primary invasive serous OvCa. Cases with borderline serous malignancies were excluded to increase etiological homogeneity. Controls (n = 445) were European women selected to be frequency matched by age with cases (by 10 year age bins). Subject ancestry was determined by self-reported four-grandparent ethnicity. Participants were not excluded based on a family history of cancer; *BRCA1* and *BRCA2* status were unknown.

### MT-DNA sequencing

Blood (90% of subjects) or saliva (10% of subjects) served as the source of genomic DNA. DNA was extracted from peripheral venous blood using a modified salting out protocol [11], and from saliva using OraGene kits (DNA Genotek, PA, USA). A panel of 64 MT-SNPs designed to tag all common European MT-DNA variation (minor allele frequency (MAF) ≥1%, *r*^2^ ≥ 0.8) [12] was genotyped in participants using the Sequenom MassARRAY iPlex Gold® system (Sequenom Inc, CA, USA) (primer sequences are available upon request). Genotyping was performed at the McGill University’s Génome Québec Innovation Centre (Montreal, Quebec) according to the manufacturer’s protocol [13]. Sequenom’s TyperAnalyzer software was used to carry out automated genotype calling. MT-SNPs were not analyzed if: (i) the call rate was <90%, (ii) they were monomorphic, or (iii) there was >1 sample with a heterozygous genotype call. Eight SNPs had a call rate <90%; 6 of these failed genotyping completely. Two SNPs were monomorphic, and 2 SNPs were heterozygous in >1 sample. Samples were excluded from analysis if the genotyping rate was less <90%. Nineteen samples had a genotyping rate <90% (13 cases, 6 controls); 12 samples failed to genotype completely (10 saliva, 2 blood). This resulted in data for 52 MT-SNPs in 831 unique samples (392 cases and 439 controls). For 44 pairs of replicate samples (5.3% replication rate) the concordance rate was 99.7%.

### MT-DNA haplogroup assignment

Samples were assigned to one of nine major European haplogroups (H, I, J, K, T, U, V, W, and X) by inspecting the alleles present at haplogroup-tagging MT-SNPs (given in Table 1). The approached used was previously described by Saxena et al., 2006. Samples that could not be unambiguously assigned a haplogroup (75 cases, 85 controls) were excluded.

**Table 1:** Association between haplogroup status and invasive serous ovarian cancer risk

**Euroeapn**	**Haplogroup-tagging SNPs**	**Allele**	**Case/control proportions**	**OR (95% CI)**	***P***
**Haplogroup**					
H	rs2015062	C	0.53 / 0.50	1.10 (0.82-1.51)	0.54
U	rs28358571, rs2853499, rs2854122	T, A, C	0.19 / 0.20	0.94 (0.64-1.39)	0.77
J	rs2015062, rs2854122, rs28359178	T, C, A	0.10 / 0.09	1.16 (0.70-1.93)	0.61
K	rs28358571	C	0.09 / 0.09	0.98 (0.59-1.65)	1.00
V	rs2015062, rs2853495, rs2853506	T, G, A	0.05 / 0.07	0.63 (0.32-1.23)	0.18
W	rs2854122, rs41535848, rs28357684	T, A, G	0.02 / 0.02	1.12 (0.41-3.49)	0.79
X	rs2853517, rs2854122, rs28357684	G, T, G	0.02 / 0.03	0.67 (0.23-1.94)	0.59
I	rs8896, rs41347846, mt14182	G, C, T	0.00 / 0.00	-	1.00
T	rs3088053, rs3926883	A, C	NA / NA	-	1.00

Table is ordered by haplogroup frequency in cases. Haplogroup tagging MT-SNPs and allele are as reported by Saxena et al., 2006. OR; odd ratio, is calculated for a total of 671 samples (317 cases and 354 controls) assigned a unique haplogroup. *P* is the Fischer’s exact P-value for testing haplogroup frequency and case status. Haplogroup T could not be assigned because rs3926883 was not genotyped. This SNP did not pass quality control. NA is not available.

### Statistical analysis

Descriptive statistics were generated using means and standard deviations for continuous variables and frequencies and percent’s for categorical variables. Distributions of covariates among cases and controls were compared using *t*-tests and χ^2^ tests of independence for continuous and categorical variables, respectively. Binary logistic regression was used to estimate the odds ratio (OR) and 95% confidence intervals (C.I.) for carriage of the minor versus major allele by case-control status. This model was adjusted for age (by 5 year age bins: <40, 40-44, 45-49, 50-54, 55-59, 60-64, 65-69, >70). Asymptotic *P*-values (*P*) and point-wise empirical *P*-values (*P*_*emp*_) (based on 10,000 permutations of case-control status) were generated to evaluate significance. Adjusting for BMI, parity, smoking history, oral contraceptive (OC) use, hormone therapy (HT) use, and a family history of ovarian or breast cancer did not influence our SNP results; therefore, all data are presented without correction for these variables. Logistic regression analyses were implement in PLINK (v1.07). MitoTool [14] was used to analyze the relationship between European haplogroup status and case-control status using Fisher’s exact test.

## Results

### Subject characteristics

Characteristics of the 392 cases and 439 controls included in the association analyses are given in Table 2. BMI, parity and a family history of breast or ovarian cancer were not significantly different between cases and control participants. Controls were more likely to have used oral contraceptives (“never used” was 37% for cases and 25% for controls, *P* = 0.0006), and more likely to have smoked (57% of cases report “none” versus 47% of controls, *P* = 0.006). Cases were more likely to have used female HT (“ever used” was 48% for cases and 40% for controls, *P* = 0.032).

**Table 2:** Select characteristics of study participants, stratified by case-control status

**Variable**	**Cases (N, %) or (Mean, SD)**	**Controls (N, %) or (Mean, SD)**
N	392	439
Age (y)	60.9 (10.3)	60.2 (10.4)
BMI^1^ (kg/m^2^)	25.3 (4.8)	25.9 (4.9)
Parity^2^		
Nulliparous	62 (16)	53 (12)
1-2 pregnancies	146 (37)	179 (41)
3+ pregnancies	184 (47)	207 (47)
Missing	0 (0)	0 (0)
OC use		
Never	146 (37)	112 (25)
Ever	245 (63)	327 (75)
Missing	1 (0)	0 (0)
Family history of ovarian or breast cancer in >1 FDR		
No	290 (74)	352 (80)
Yes	87 (22)	75 (17)
Missing	15 (4)	12 (3)
Pack-years smoked^3^		
None	224 (57)	206 (47)
< 20	68 (17)	118 (27)
> 20	98 (25)	113 (26)
Missing	2 (0)	2 (0)
HT use		
Never	205 (52)	263 (60)
Ever	187 (48)	176 (40)
Missing	0 (0)	0 (0)

Percentages are rounded and may not add up to 100. Abbreviations used: N, number of observations; SD, standard deviation; OC, oral contraceptive; HT, hormone therapy; FDR, first degree relative.

^1^BMI was calculated using usual adult or current weight for participants.

^2^Parity was based on the number of births, regardless of outcome.

^3^Where “None” is < 100 cigarettes during a lifetime.

### SNP and haplogroup association results

Of the 52 MT-SNPs tested for association with case-control status, the most significant result was for a synonymous MT-SNP (rs2857285) in *ND4* (OR = 4.84, 95% CI: 1.03–22.68, *P* = 0.045, *P*_*emp*_ *=* 0.037) (Additional file 1: Table S1). After adjustment for multiple testing using a Bonferroni correction of the Type 1 error this MT-SNP was not significant. rs2857285 was relatively uncommon, detected in 2.3% of cases and 0.5% of controls. No other SNP tested had a P-value < 0.05. Gene-wide tests of association were not performed on our MT-SNP data because of the lack of a significant result, or a result “suggestive” of significance, in the individual SNP-specific tests of association.

Among our study participants, 19% (10/52) of SNPs qualified as 'rare’ (MAF <0.01 in both cases and controls), and 54% (28/52) had a MAF < 5% (in both cases and controls). Thus, for most SNPs tested we were only powered to detect large effects (OR > 2). Association results for MT-SNPs with MAF > 5% are given in Table 3 (results for all MT-SNPs are given in Additional file 1: Table S1).

**Table 3:** MT-SNP-invasive serous ovarian cancer risk association results (MT-SNPs MAF >5%)

**SNP**	**ID**	**BP**	**Major/minor allele**	**Case/control MAF**	**N**	**OR (95% CI)**	***P***	***P***_***emp***_
rs2853517	mt709	709	G/A	0.167 / 0.153	830	1.10 (0.76-1.60)	0.61	0.86
rs41352944	mt930	930	C/T	0.056 / 0.043	831	1.30 (0.69-2.46)	0.41	0.55
rs28358571	mt1189	1189	T/C	0.077 / 0.077	831	0.98 (0.59-1.64)	0.94	1.00
rs3928306	mt3010	3010	C/T	0.209 / 0.212	831	1.01 (0.72-1.41)	0.96	1.00
rs2015062	mt7028	7028	A/G	0.408 / 0.428	831	0.93 (0.70-1.23)	0.60	0.64
rs2853825	mt9477	9477	G/A	0.105 / 0.084	830	1.27 (0.80-2.04)	0.31	0.38
rs2853495	mt11719	11719	A/G	0.457 / 0.456	831	1.01 (0.76-1.33)	0.97	1.00
rs3088053	mt11812	11812	A/G	0.079 / 0.075	831	1.06 (0.63-1.78)	0.82	0.73
rs2853499	mt12372	12372	C/T	0.249 / 0.239	825	1.05 (0.76-1.45)	0.77	0.86
rs2854122	mt12705	12705	G/A	0.097 / 0.097	795	1.01 (0.63-1.62)	0.97	1.00
rs2853504	mt14793	14793	T/C	0.054 / 0.055	831	1.00 (0.54-1.82)	0.99	1.00
rs28357681	mt14798	14798	A/G	0.154 / 0.169	829	0.90 (0.62-1.31)	0.59	0.73
rs28357684	mt15043	15043	G/A	0.064 / 0.062	829	1.06 (0.60-1.88)	0.83	0.86
rs2853510	mt15924	15924	A/G	0.066 / 0.068	831	0.97 (0.56-1.68)	0.91	1.00

Sorted by mitochondrial base-pair position. ID is SNP as reported by Saxena et al., 2006, available at: http://www.broadinstitute.org/mpg/tagger/mito.html [Accessed 15 Nov, 2012]. BP, mitochondrial base-pair position according to the revised Cambridge reference sequence; MAF, minor allele frequency; N, number of observations; OR, odds ratio; 95% CI, 95% confidence interval. OR and 95% CI are for logistic regression adjusting for age at diagnosis or interview. *P* is the asymptotic *P*-value for testing MT-SNP effect significance; P_*emp*_ is empirical point-wide P-value, implement in PLINK (v1.07).

We did not detect a significant relationship between European MT haplogroup (H, U, J, V, K, W, X, I) status and case status (Table 1). To assess whether MT-SNP association results were influenced by haplogroup background, we also performed a logistic regression adjusting for age and haplogroup. No SNP had a *P*-value < 0.05 under this model (data not shown).

## Discussion

This report explored the hypothesis that inherited MT-SNPs and/or haplogroup influence invasive serous OvCa risk. The analysis focused on a panel of MT-SNPs previously described to tag all common variants observed in the European population (MAF > 1%, r^2^ > 0.8), and was intended to exhaustively examine the entire MT genome for association with invasive serous OvCa risk. For the 52 MT-SNPs tested we found little evidence of association with invasive serous OvCa risk; however, because 12 MT-SNPs were not examined (having failed genotyping) our analysis was not as exhaustive as intended. We found no association between European MT haplogroup and invasive serous OvCa risk.

We found that most MT-SNPs (73%) were relatively uncommon (MAF <5%); thus, only those conferring large OvCa risk (OR > 2) would be reliably detected by our study, and these do not appear to exist. The MAF estimates observed in our study are consistent with previous studies performed in Europeans using this SNP panel [12,15]. A larger sample size is needed to test these lower MAF MT-SNPs for association with invasive serous OvCa risk. For example, approximately 5000 cases and controls would be needed to detect an OR of 2.0 for MT-SNPs with a MAF around 1%.

Two previous studies have examined the association between inherited variation in the MT genome and OvCa risk. The first was a small study performed on women of Chinese descent [10], and tested 1 MT-DNA SNP (16189T > C) for association with OvCa risk in 53 cases (histological subtype not specified) and 107 controls. No association was detected. The second was a much larger study performed in on Caucasian women [9], and tested 128 MT-SNPs for association with OvCa risk in 1,815 cases (histological subtypes combined) and 1,900 controls. The MT-SNPs tested were those found on Illumina’s Infinium 610K beadchip, a commercially available array designed for whole-genome genotyping. In this study, one MT-SNP in the gene *MT-CO1 (COX1,* T6777C), was reported to decrease OvCa risk (OR = 0.68, 95% CI: 0.51-0.92, *P =* 0.006). Unfortunately, this SNP failed to genotype; thus, we are unable to provide additional evidence for or against its association with OvCa risk. The most associated MT-SNP reported by our study, rs2857285, was not genotyped by Permuth-Wey et al. [9]; hence, additional information on this SNP is not available from this source.

The greatest strength of this study was the population-based collection of European women with recently collected, detailed epidemiological data, and available high quality DNA samples. This study’s greatest limitation was power. Due to small sample size this study was only powered to reliably detect moderate to large MT-SNP effect sizes. MT-SNPs associated with small effects on OvCa risk, and MT-SNPs with low MAF would not be reliably detected. A further limitation was the potential for population stratification given principal components (PCs) representing European ancestry were not available for all study participants and were not included in our analyses. Subsequent, in an independent study 50% of our participants were confirmed to be of >90% European ancestry using the program LAMP (Pharoah et al., in submission). Although the remaining 50% were not examined, we see no reason why their ancestry would differ. When individuals with PCs (N = 408) were analyzed adjusting for the first 5 eigenvalues from the PC analysis, no MT-SNP-cancer associations had a *P*-value <0.05. Thus, we do not anticipate that inclusion of PC’s would change the conclusion of this study.

## Conclusion

There is relatively little evidence that inherited MT-SNP variation contributes to invasive serous OvCa risk in European women. Conceivably, rare MT-SNP variants (MAF <1%) conferring large OvCa risk account for a portion of the heritable risk of this disease. A full genome sequencing approach in a large study cohort could explore this possibility. Furthermore, these studies have the potential to identify indels and heteroplasmy, which have yet to be examined with respect to heritable OvCa risk.

## Competing interests

The authors declare no competing interests.

## Authors’ contributions

ME carried out the statistical analysis and drafted the manuscript. ABW, NL, and LC conceived the study. All authors read and approved the manuscript.

## Supplementary Material

Additional file 1: Table S1 MT-SNP-invasive serous ovarian cancer risk association results. Click here for file
