# Supplementary material for: Inherited common variants in mitochondrial DNA and invasive serous epithelial ovarian cancer risk
Source: BMC Res Notes. 2013 Oct 22;6:425. doi: 10.1186/1756-0500-6-425 (PMC3854008; doi:10.1186/1756-0500-6-425)
Supplement: Additional file 1: Table S1 — MT-SNP-invasive serous ovarian cancer risk association results. [file 1756-0500-6-425-S1.docx]

**Table S1.** MT-SNP-invasive serous ovarian cancer risk association results.

| # | Rs number | Alt_ID | BP | Maj/Min | F_A | F_U | N | OR | L95 | U95 | *P* | *P_emp_* |
| --- | --- | --- | --- | --- | --- | --- | --- | --- | --- | --- | --- | --- |
| 1 | rs2853517 | mt709 | 709 | G/A | 0.166 | 0.153 | 830 | 1.10 | 0.76 | 1.60 | 0.61 | 0.86 |
| 2 | rs2853518 | mt750 | 750 | G/A | 0.015 | 0.011 | 831 | 1.32 | 0.40 | 4.38 | 0.65 | 0.86 |
| 3 | rs41352944 | mt930 | 930 | C/T | 0.056 | 0.043 | 831 | 1.30 | 0.69 | 2.46 | 0.41 | 0.55 |
| 4 | rs28358571 | mt1189 | 1189 | T/C | 0.077 | 0.077 | 831 | 0.98 | 0.59 | 1.64 | 0.94 | 1.00 |
| 5 | rs3928306 | mt3010 | 3010 | C/T | 0.209 | 0.212 | 831 | 1.01 | 0.72 | 1.41 | 0.96 | 1.00 |
| 6 | rs41460449 | mt3394 | 3394 | A/G | 0.013 | 0.011 | 830 | 1.14 | 0.32 | 4.03 | 0.84 | 0.86 |
| 7 | rs41524046 | mt3915 | 3915 | G/A | 0.038 | 0.025 | 831 | 1.54 | 0.69 | 3.40 | 0.29 | 0.36 |
| 8 | rs41456348 | mt4336 | 4336 | T/C | 0.013 | 0.021 | 828 | 0.63 | 0.21 | 1.92 | 0.42 | 0.45 |
| 9 | rs3021086 | mt4769 | 4769 | G/A | 0.023 | 0.018 | 830 | 1.30 | 0.49 | 3.42 | 0.59 | 0.64 |
| 10 | none | mt4793 | 4793 | T/C | 0.013 | 0.025 | 827 | 0.47 | 0.16 | 1.39 | 0.17 | 0.16 |
| 11 | none | mt5426 | 5426 | T/C | 0.010 | 0.018 | 831 | 0.55 | 0.16 | 1.86 | 0.33 | 0.27 |
| 12 | rs3902405 | mt5465 | 5465 | A/G | 0.000 | 0.002 | 831 | - | - | - | - | - |
| 13 | none | mt5656 | 5656 | T/C | 0.018 | 0.018 | 831 | 0.93 | 0.33 | 2.61 | 0.89 | 1.00 |
| 14 | rs28623747 | mt6260 | 6260 | C/T | 0.010 | 0.018 | 831 | 0.52 | 0.16 | 1.76 | 0.30 | 0.27 |
| 15 | rs41464546 | mt6365 | 6365 | T/C | 0.003 | 0.002 | 830 | 1.35 | 0.08 | 22.32 | **0.84** | **0.05** |
| 16 | rs2015062 | mt7028 | 7028 | A/G | 0.408 | 0.428 | 831 | 0.93 | 0.70 | 1.23 | 0.61 | 0.64 |
| 17 | rs8896 | mt8269 | 8269 | G/A | 0.013 | 0.018 | 831 | 0.72 | 0.23 | 2.23 | 0.56 | 0.48 |
| 18 | none | mt8705 | 8705 | A/G | 0.005 | 0.002 | 831 | 2.14 | 0.19 | 23.92 | 0.54 | 0.23 |
| 19 | none | mt8869 | 8869 | T/C | 0.008 | 0.000 | 828 | - | - | - | - | - |
| 20 | rs28358270 | mt9123 | 9123 | C/T | 0.010 | 0.014 | 831 | 0.78 | 0.22 | 2.79 | 0.70 | 0.78 |
| 21 | none | mt9150 | 9150 | A/G | 0.015 | 0.007 | 830 | 2.40 | 0.59 | 9.75 | 0.22 | 0.28 |
| 22 | rs2853825 | mt9477 | 9477 | G/A | 0.105 | 0.084 | 830 | 1.27 | 0.80 | 2.04 | 0.31 | 0.39 |
| 23 | rs41482146 | mt9667 | 9667 | A/G | 0.013 | 0.009 | 831 | 1.44 | 0.38 | 5.46 | 0.59 | 0.45 |
| 24 | rs41502750 | mt9716 | 9716 | A/G | 0.023 | 0.023 | 831 | 1.01 | 0.40 | 2.51 | 0.99 | 0.78 |
| 25 | rs41345446 | mt9899 | 9899 | T/C | 0.021 | 0.032 | 827 | 0.65 | 0.27 | 1.59 | 0.35 | 0.50 |
| 26 | rs41487950 | mt10084 | 10084 | A/G | 0.008 | 0.005 | 822 | 1.61 | 0.27 | 9.74 | 0.61 | 0.48 |
| 27 | rs2857285 | mt10915 | 10915 | T/C | 0.023 | 0.005 | 830 | 4.84 | 1.03 | 22.68 | 0.045* | 0.037* |
| 28 | rs41537746 | mt11377 | 11377 | C/T | 0.015 | 0.034 | 831 | 0.41 | 0.16 | 1.09 | 0.07 | 0.06 |
| 29 | rs28358286 | mt11674 | 11674 | G/A | 0.020 | 0.021 | 831 | 0.96 | 0.37 | 2.53 | 0.94 | 1.00 |
| 30 | rs2853495 | mt11719 | 11719 | A/G | 0.457 | 0.456 | 831 | 1.01 | 0.76 | 1.33 | 0.97 | 1.00 |
| 31 | rs3088053 | mt11812 | 11812 | A/G | 0.079 | 0.075 | 831 | 1.06 | 0.64 | 1.78 | 0.82 | 0.73 |
| 32 | rs2853496 | mt11914 | 11914 | G/A | 0.031 | 0.018 | 829 | 1.70 | 0.68 | 4.23 | 0.26 | 0.27 |
| 33 | rs2853497 | mt12007 | 12007 | G/A | 0.005 | 0.018 | 830 | 0.28 | 0.06 | 1.35 | 0.11 | 0.09 |
| 34 | rs2853499 | mt12372 | 12372 | C/T | 0.249 | 0.239 | 825 | 1.05 | 0.76 | 1.45 | 0.78 | 0.86 |
| 35 | rs41520546 | mt12414 | 12414 | A/G | 0.018 | 0.021 | 831 | 0.85 | 0.31 | 2.32 | 0.75 | 0.86 |
| 36 | rs2854122 | mt12705 | 12705 | G/A | 0.097 | 0.097 | 795 | 1.01 | 0.63 | 1.62 | 0.97 | 1.00 |
| 37 | none | mt13020 | 13020 | T/C | 0.010 | 0.011 | 830 | 0.86 | 0.23 | 3.30 | 0.83 | 1.00 |
| 38 | rs2853501 | mt13105 | 13105 | T/C | 0.005 | 0.005 | 830 | 1.11 | 0.15 | 7.98 | 0.92 | 0.78 |
| 39 | none | mt13879 | 13879 | A/G | 0.003 | 0.005 | 826 | 0.49 | 0.04 | 5.55 | 0.56 | 0.52 |
| 40 | rs41509754 | mt13965 | 13965 | T/C | 0.005 | 0.018 | 828 | 0.27 | 0.06 | 1.29 | 0.10 | 0.09 |
| 41 | rs41535848 | mt13966 | 13966 | T/C | 0.023 | 0.025 | 831 | 0.95 | 0.39 | 2.34 | 0.92 | 1.00 |
| 42 | rs3135030 | mt14470 | 14470 | A/G | 0.021 | 0.016 | 822 | 1.40 | 0.50 | 3.97 | 0.52 | 0.50 |
| 43 | rs2853504 | mt14793 | 14793 | T/C | 0.054 | 0.055 | 831 | 1.00 | 0.54 | 1.82 | 0.99 | 1.00 |
| 44 | rs28357681 | mt14798 | 14798 | A/G | 0.154 | 0.169 | 829 | 0.90 | 0.62 | 1.31 | 0.59 | 0.73 |
| 45 | rs28357684 | mt15043 | 15043 | G/A | 0.064 | 0.062 | 829 | 1.06 | 0.60 | 1.88 | 0.84 | 0.86 |
| 46 | rs2853506 | mt15218 | 15218 | T/C | 0.033 | 0.043 | 831 | 0.77 | 0.38 | 1.59 | 0.48 | 0.55 |
| 47 | rs41518645 | mt15257 | 15257 | C/T | 0.020 | 0.025 | 831 | 0.77 | 0.30 | 1.93 | 0.57 | 0.55 |
| 48 | rs41337244 | mt15758 | 15758 | T/C | 0.018 | 0.023 | 831 | 0.77 | 0.29 | 2.07 | 0.61 | 0.52 |
| 49 | rs28357375 | mt15784 | 15784 | A/G | 0.005 | 0.007 | 828 | 0.74 | 0.12 | 4.54 | 0.75 | 0.64 |
| 50 | rs41504845 | mt15833 | 15833 | G/A | 0.011 | 0.012 | 808 | 0.98 | 0.26 | 3.71 | 0.97 | 1.00 |
| 51 | rs28617642 | mt15884 | 15884 | G/A | 0.003 | 0.014 | 814 | 0.18 | 0.02 | 1.52 | 0.12 | 0.07 |
| 52 | rs2853510 | mt15924 | 15924 | A/G | 0.066 | 0.068 | 831 | 0.97 | 0.56 | 1.68 | 0.91 | 1.00 |

Sorted by mitochondrial base-pair position. Alternate ID is as reported by Saxena et al., 2006, available at: http://www.broadinstitute.org/mpg/tagger/mito.html [Accessed 15 Nov, 2012]. BP, mitochondrial base-pair position according to the revised Cambridge reference sequence; Maj/min, major and minor allele; F_A, minor allele frequency in cases; F_A, minor allele frequency in controls; N, number of observations; OR, odds ratio; 95%CI, 95% confidence interval. OR and 95% CI are for logistic regression adjusting for age at diagnosis or interview. *P* is the asymptotic *P*-value for testing MT-SNP effect significance; P*_emp_* is empirical point-wide P-value, implement in PLINK (v1.07). Asterisks indicate significant P-values.
